# Graph-based analysis of brain connectivity in schizophrenia

**DOI:** 10.1371/journal.pone.0188629

**Published:** 2017-11-30

**Authors:** Elzbieta Olejarczyk, Wojciech Jernajczyk

**Affiliations:** 1 Nalecz Institute of Biocybernetics and Biomedical Engineering, Polish Academy of Sciences, Warsaw, Poland; 2 Department of Clinical Neurophysiology, Institute of Psychiatry and Neurology, Warsaw, Poland; University of Electronic Science and Technology of China, CHINA

## Abstract

The present study evaluated brain connectivity using electroencephalography (EEG) data from 14 patients with schizophrenia and 14 healthy controls. Phase-Locking Value (PLV), Phase-Lag Index (PLI) and Directed Transfer Function (DTF) were calculated for the original EEG data and following current source density (CSD) transformation, re-referencing using the average reference electrode (AVERAGE) and reference electrode standardization techniques (REST). The statistical analysis of adjacency matrices was carried out using indices based on graph theory. Both CSD and REST reduced the influence of volume conducted currents. The largest group differences in connectivity were observed for the alpha band. Schizophrenic patients showed reduced connectivity strength, as well as a lower clustering coefficient and shorter characteristic path length for both measures of phase synchronization following CSD transformation or REST re-referencing. Reduced synchronization was accompanied by increased directional flow from the occipital region for the alpha band. Following the REST re-referencing, the sources of alpha activity were located at parietal rather than occipital derivations. The results of PLV and DTF demonstrated group differences in fronto-posterior asymmetry following CSD transformation, while for PLI the differences were significant only using REST. The only analysis that identified group differences in inter-hemispheric asymmetry was DTF calculated for REST. Our results suggest that a comparison of different connectivity measures using graph-based indices for each frequency band, separately, may be a useful tool in the study of disconnectivity disorders such as schizophrenia.

## Introduction

Schizophrenia is considered as a disorder of brain disconnectivity [1–2], characterized by profound disruption of large-scale prefronto-temporal interactions [3]. Animal models show that developmental hippocampal lesions may cause disconnectivity of the prefrontal cortex [1]. Magnetic resonance imaging (MRI) studies in patients with schizophrenia have revealed gray matter volume deficits in the medial temporal lobe (including hippocampus) and in the heteromodal association cortex (including the prefrontal, anterior cingulate, superior temporal and parietal cortex, and the thalamus), as well as enlargement of the ventricles [4]. Diffusion tensor imaging (DTI) studies have shown decreased white matter anisotropy in the deep left prefrontal and temporal cortex, and reductions in the myelin membranes [5]. The limbic pathways connecting the hippocampus with the prefrontal cortex, anterior cingulate cortex, and thalamus are involved in higher cognition and information processing [6]. Thus, one consequence of abnormal connectivity in schizophrenia may be a loss of coherent functional integration that results in a disorder of association processes.

The cortical disconnectivity hypothesis was also investigated by the analysis of neurophysiological data. The analysis of electroencephalography (EEG) and magnetoencephalography (MEG) signals, due to their high temporal resolution, provides unique information regarding neuronal oscillations and cross-frequency coupling, i.e. interactions between different frequency bands [7–9]. These interactions have been shown to be impaired in schizophrenia [10]. The analysis of EEG during a resting state condition showed increased delta, theta, and beta activity, as well as decreased activity in alpha frequency in the frontal cortex (“*hypofrontality*”) in patients relative to controls [11–12]. Another feature of the brain, which has emerged as an attractive potential biomarker of schizophrenia, is *abnormal brain asymmetry* [13–16]. The hemispheres of the human brain are anatomically and functionally asymmetric and many cognitive and motor functions are lateralized. For example, in healthy persons, language processing is lateralized to the left hemisphere of the brain. Such leftward cerebral dominance is reduced in patients with schizophrenia, which may result in language impairment [17–19]. DTI studies have shown structural reductions in some key white matter tracts (e.g. corpus callosum), which may contribute to abnormal intra-hemispheric connectivity in schizophrenia [15–16], as well as impaired transfer of information from the right to the left hemisphere [20]. Moreover, hemispheric asymmetry is associated with a reduction of gray matter in frontal and temporal areas [21]. Although the mechanism underlying left-hemisphere dominance is presently unknown, studies suggest a role for early disturbance in neuronal development. It has even been suggested that language and psychosis have a common evolutionary origin, and that schizophrenia may be related to genetic variation occurring during evolution of hemispheric dominance for language in *Homo sapiens* [22–23]. The pattern of functional asymmetry in schizophrenia may differ between patients suffering from positive and negative symptoms. While positive symptoms are associated with an increase of leftward asymmetry, negative symptoms are associated with an increase of rightward asymmetry of functional connectivity [24]. Other studies report an association between increased activation in the right hemisphere and auditory hallucinations (e.g. perceiving one's own thoughts as an external voice) and delusions [25–26].

Interactions between functionally specialized brain areas can be described by means of different connectivity measures and indices based on graph theory. There are three classes of connectivity: anatomical (or structural), functional, and effective [27]. Anatomical connectivity is defined as the network of physical connections between brain structures. Functional connectivity refers only to statistical dependences between these structures, while effective connectivity provides information about the causal interactions between (or among) them. The statistical significance of these measures can be evaluated using indices based on graph theory. In graph theory, the brain is modelled as a graph composed of nodes that represent EEG channels, and links that represent the connectivity between them. In contrast to random networks, normal brain network organization is characterized by small-worldness, e.g., shorter characteristic path lengths and higher local clustering (existence of modules and high-degree nodes, i.e. hubs). Such network architecture enables optimal information processing and network robustness. Another important characteristic of normal brain network organization is hierarchy, which is reflected in a fractal nature of network organization, i.e., existence of self-similarity pattern at every spatial and temporal scale, as well as different structural and functional organization levels [28–34]. This network architecture, expressed by structural and functional connectivity, is disrupted in some neuropsychiatric disorders such as schizophrenia (reviewed by [35–36]). Network abnormalities exhibit topological patterns specific to each disorder. In schizophrenia, the gray matter lesions are concentrated in hubs located in the frontal and temporal lobes.

In the present study, we compare three connectivity measures in schizophrenia patients and healthy controls: Phase-Locking Value (PLV), Phase-Lag Index (PLI), and Directed Transfer Function (DTF). The PLV and PLI allow for quantification of phase synchronization. The DTF, in contrast to the PLV and PLI, is a directional connectivity measure, which provides information about the causal interactions between different brain structures. The statistical significance of the above connectivity measures was evaluated using indices based on graph theory. The choice of reference electrode may have an impact on the connectivity analysis [37–42]. Thus, we compared our results obtained using the scalp EEG data with results obtained using data that was re-referenced using the average reference electrode (AVERAGE) [43], scalp surface Laplacian or current source density (CSD) analysis [44–46] and reference electrode standardization techniques (REST) [37, 47–48].

## Materials and methods

### Participant recruitment

The study comprised of 14 patients (7 males: 27.9 ± 3.3 years, 7 females: 28.3 ± 4.1 years) with paranoid schizophrenia, who were hospitalized at the Institute of Psychiatry and Neurology in Warsaw, Poland, and 14 healthy controls (7 males: 26.8 ± 2.9, 7 females: 28.7 ± 3.4 years). The patients met International Classification of Diseases ICD–10 criteria for paranoid schizophrenia (category F20.0). Study protocol was approved by the Ethics Committee of the Institute of Psychiatry and Neurology in Warsaw. All participants received a written description of the protocol and provided written consent to take part in this study. Inclusion criteria were: a minimum age of 18, ICD-10 diagnosis F20.0, and medication washout period of a minimum of seven days. Exclusion criteria were: pregnancy, organic brain pathology, severe neurological diseases (e.g. epilepsy, Alzheimer’s, or Parkinson disease), presence of a general medical condition, and very early stage of schizophrenia, i.e., first episode of schizophrenia. The control group was matched in gender and age to the 14 patients completing the study.

### EEG recording and preprocessing

Fifteen minutes of EEG data were recorded in all subjects during an eyes-closed resting state condition. Data were acquired with the sampling frequency of 250 Hz using the standard 10–20 EEG montage with 19 EEG channels: Fp1, Fp2, F7, F3, Fz, F4, F8, T3, C3, Cz, C4, T4, T5, P3, Pz, P4, T6, O1, O2. The reference electrode was placed at FCz. EEG analysis was carried out using thirty second segments without artefacts (i.e. eye movements, cardiac activity, muscle contractions). Then, the signals of each EEG channel were filtered using a Butterworth filter of order 2 in the following physiological frequency bands: 2–4 Hz (delta), 4.5–7.5 Hz (theta), 8–12.5 Hz (alpha), 13–30 Hz (beta), 30–45 Hz (gamma).

EEG analysis was carried out using the raw EEG data, as well as the re-referenced data relative to scalp surface Laplacian estimators (commonly referred to as current source density (CSD) [44–46]), the average reference electrode (AVERAGE) [43], and the reference electrode standardization techniques (REST) [37, 47–48]. This permitted us to examine the potential effect of reference electrode choice on connectivity results.

CSD transformation was calculated using the CSD Toolbox, which allows for estimation of surface potentials using a spherical spline algorithm [49]. REST was carried out using the REST Toolbox (http://www.neuro.uestc.edu.cn/rest/). REST is a method that allows for the transformation of original EEG data—with the reference electrode placed at an arbitrary point on the head—to a new dataset with the reference at infinity and the potential at zero or a constant. The procedure is based on the calculation of the leadfield matrix for the canonical concentric-three-spheres head model.

The current widely recommended reference is the REST. It was shown that the relative error of REST is always smaller than AVERAGE and it decreases by increasing the number of channels in contrast to the error of AVERAGE [50–51]. Moreover, considering that the CSD transform is free from reference effects, we expected a greater correlation between CSD and REST than between CSD and another reference electrode.

### Connectivity measures

EEG data were analyzed using three connectivity methods: Phase-Locking Value, Phase-Lag Index, and Directed Transfer Function, as well as indices based on graph theory. Below, is the brief theoretical basis for each method.

#### Phase-locking value

The amplitude A(t) and the instantaneous phase Ф (t) of a signal s(t) can be estimated using the Hilbert transform H{·} [52–53]:

$$z(t)=s(t)+i\cdot H\left\{s(t)\right\}=A(t)\cdot e^{i\cdot \phi (t)}$$ (Eq 1)

The analytic signal z(t) can be understood as an embedding of the one dimensional time-series in the two dimensional complex plane.

The phase is computed by the following expression:

ϕ(t)=arctan(Im{z(t)}Re{z(t)})=arctan(H{s(t)}s(t));ϕ∈[−π,π] (Eq 2)

The phase synchronization is defined as the locking of phases of two oscillators:

$$\phi_{12}(t)=\phi_{1}(t)-\phi_{2}(t)=const.$$ (Eq 3)

where Φ_1_(t) and Φ_2_(t) denote the phases of the oscillators, and Φ_12_(t) is defined as their relative phase.

The phase-locking value (PLV) is defined as:

$$PLV=\left|\frac{1}{N}\sum_{j=0}^{N-1}e^{i\phi_{12}(j\Delta t)}\right|={\left({\left[\frac{1}{N}\sum_{j=0}^{N-1}\sin(\phi_{12}(j\Delta t))\right]}^{2}+{\left[\frac{1}{N}\sum_{j=0}^{N-1}\cos(\phi_{12}(j\Delta t))\right]}^{2}\right)}^{1/2}$$ (Eq 4)

where

i–imaginary unit; N–the total number of samples; Φ_12_ –the relative phase of two signals; Δt–time between the successive samples j from 1 to N-1.

The value of PLV is bounded between zero and one with zero indicating unsynchronized phases and one indicating when the phase difference is constant, i.e. the synchronization of signals is perfect. A decrease of the phase-locking value between two signals indicates a loss of synchronization between them.

#### Phase-Lag Index

Phase-Lag Index (PLI) was introduced to reduce the impact of common sources (e.g., volume conduction and/or active reference electrodes in the case of EEG) in the estimation of phase synchronization [54].

An asymmetry index for the distribution of phase differences centered around 0 mod π, is defined as:

$$PLI=\left|\frac{1}{N}\sum_{n=1}^{N}sign(\Delta \Phi (t_{n}))\right|$$ (Eq 5)

where–π < ΔΦ(t_n_) ≤ π, n = 1…N is a time series of phase differences.

The PLI ranges between zero and one, with zero indicating either no coupling or coupling with a phase difference centered around 0 mod π, and one indicating perfect phase locking at a value of ΔΦ different from 0 mod π.

#### Directed Transfer Function

Directed Transfer Function (DTF) is a measure based on Granger Causality, but defined in the frequency domain [55]:

For a multivariate k-channel process: **X**(t) = (X_1_(t), X_2_(t),…, X_k_(t)), the multivariate autoregressive model takes the form:

$$X(t)=\sum_{m=1}^{p}\hat{A}(m)\cdot X(t-m)+E(t)\;or\;\sum_{m=0}^{p}A(m)\cdot X(t-m)=E(t)$$ (Eq 6)

where **E**(t) is a k–dimensional vector, $\hat{A}$ is a square k x k matrix.

Transforming the multivariate autoregressive model to the frequency domain we obtain:

$$A(f)X(f)=E(f),\;where\;A(f)=-\sum_{m=1}^{k}A(m)\cdot e^{-i\cdot 2\pi \cdot f\cdot m}\to \;X(f)=A^{-1}(f)E(f)=H(f)E(f)$$ (Eq 7)

The matrix of coefficients **H** is called the transfer matrix.

The Directed Transfer Function is defined as a normalized version of the transfer matrix:

$$DTF^{2}{}_{j\to i}(f)=\frac{{\left|H_{ij}(f)\right|}^{2}}{\sum_{j=1}^{k}{\left|H_{ij}(f)\right|}^{2}}$$ (Eq 8)

The following relation should be satisfied to guarantee the quality of fitting of the model [56]:

k⋅d<0.1⋅N (Eq 9)

where N is the window length; d is the model order; and k is the number of EEG channels. The model order was estimated using the Akaike information criterion [57]. In this paper the model order d was set to 10.

#### Graph analysis

All of the measures (PLV, PLI, DTF) were calculated from 30-second EEG epochs subdivided into three consecutive 10-second trials. For each connectivity measure, matrices of significant connections (the so-called adjacency matrices) were produced for each frequency band separately, as well as for whole spectrum, and used as weighted adjacency matrices. To assure the correct estimation of the autoregressive model, the DTF was calculated in the whole frequency band, and then the adjacency matrices were identified for individual frequency bands separately. The adjacency matrices were analyzed using indices based on graph theory [58–59]. In graph theory, the brain is modeled as a graph composed of nodes, representing brain regions or EEG channels, and undirected or directed links between them, representing functional connections determined by PLV, PLI and DTF. The connectivity measures were calculated using algorithms included in the MATLAB Toolbox—HERMES (http://hermes.ctb.upm.es) [60], while the graph-based indices were calculated using MATLAB functions embedded in the Brain Connectivity Toolbox (http://www.brain-connectivity-toolbox.net). For each of the obtained graphs, the following indices were calculated [58–59]: (1) basic measures (i.e., density, degree, and strength); (2) measure of integration (i.e., characteristic path length); (3) measure of segregation (i.e., clustering coefficient). Additionally, measures of asymmetry (i.e., D_FP_, I_FP_, D_LR_, I_LR_) were calculated.

The degree of an individual node is equal to the number of links connected to that node, which, in practice, is also equal to the number of neighbors of the node. Individual values of the degree therefore reflect the relative importance of a node in the network. The mean network degree is commonly used as a measure of the graph density, or of the total “wiring cost” of the network. The directed variant of the degree distinguishes the number of inward links from the number of outward links, while the weighted variant of the degree, termed the strength, is defined as the sum of all neighboring link weights.

Measures of integration estimate the ease of communication between distributed nodes. Specifically, the characteristic path length is defined as the average shortest path length between all pairs of nodes in the network.

Measures of segregation characterize the ability for specialized processing within densely interconnected groups of nodes (clusters or modules). The clustering coefficient is defined as the fraction of the node's neighbors that are also neighbors of each other.

Displacement and influence were introduced to analyze asymmetry between frontal and posterior parts of brain, as well as between left and right hemispheres.

These measures are defined as follows:

$$D_{FP}=\frac{k_{F}-k_{P}}{k/2\cdot \left(k/2-1\right)}$$ (Eq 10)

$$I_{FP}=\frac{k_{P\to F}-k_{F\to P}}{{\left(k/2\right)}^{2}}$$ (Eq 11)

where D–displacement; I–influence; k–number of all EEG channels, i.e. the number of rows or columns in the connectivity matrix; k_F_−number of connections within the frontal half of the brain (the first k/2 rows and the first k/2 columns of connectivity matrix); k_P_−number of connections within the posterior half of the brain (the last k/2 rows and the last k/2 columns); kP→F−number of connections from posterior to frontal areas of the brain (the first k/2 rows and the last k/2 columns of connectivity matrix); k_F→P_−number of connections from frontal to posterior areas of the brain (the last k/2 rows and the first k/2 columns of connectivity matrix).

By analogy, the measures of asymmetry between the left and right hemispheres (D_LR_, I_LR_) are defined.

#### Statistical analysis

In the adjacency matrix, only the connectivity values above a significance threshold of 0.05 were considered, while all non-significant values were set to zero. The significance of the connectivity values was determined by surrogate data analysis [61]. The surrogate data method involves maintaining the amplitudes while destroying the phase relationship between the original signals by randomly shuffling the data in the frequency domain and then transforming them back to the time domain. The significance was tested by comparing values from the original data to values obtained from surrogate data. The number of surrogates, which were calculated for each signal, was 100.

To evidence different connectivity patterns between the two groups (i.e., healthy persons and patients with schizophrenia) a three-way ANOVA model was separately applied to the following indices for each metric: degree and strength. The ANOVA had the following factors: *Group*, *Band* (delta; theta; alpha; beta; gamma), and *EEG channels* (system of 19 channels: Fp1, Fp2, F7, F3, Fz, F4, F8, T3, C3, Cz, C4, T4, T5, P3, Pz, P4, T6, O1, and O2). Moreover, a two-way ANOVA model with variables: *Group* and *EEG channels*, was analyzed for the same indices in each band separately. A two-way ANOVA model with variables: *Group* and *Band*, was analyzed for density, characteristic path length, clustering coefficient, displacement and influence.

In case of significant effects, *post hoc* tests (Bonferroni correction) were performed. The statistical threshold was set at p<0.05, with correction for multiple comparisons by controlling the family wise error (FWE).

## Results

**Fig 1:**
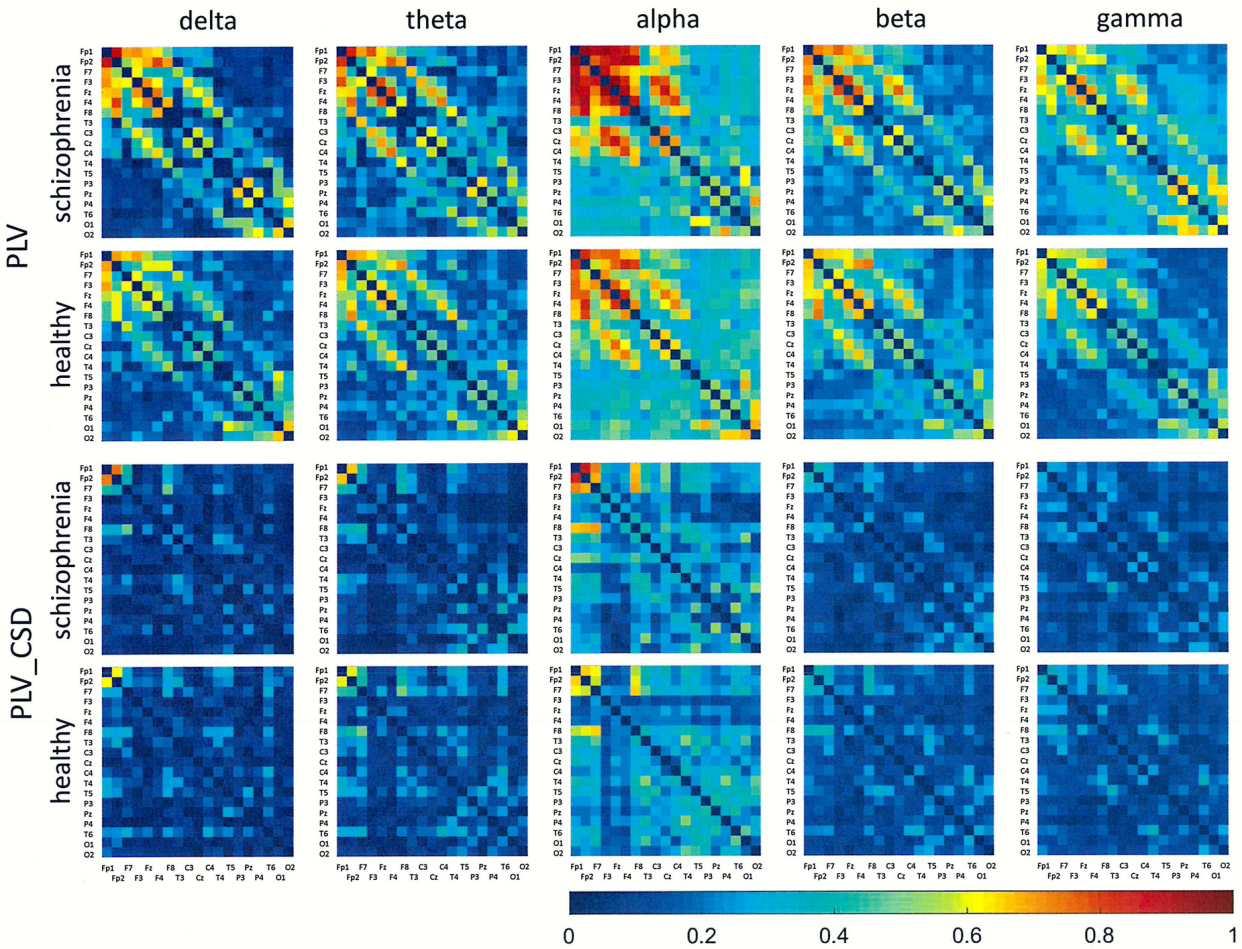
Mean adjacency matrices for PLV in patients with schizophrenia and healthy controls for delta, theta, alpha, beta, and gamma frequency bands using FCz or CSD as a reference.

**Fig 2:**
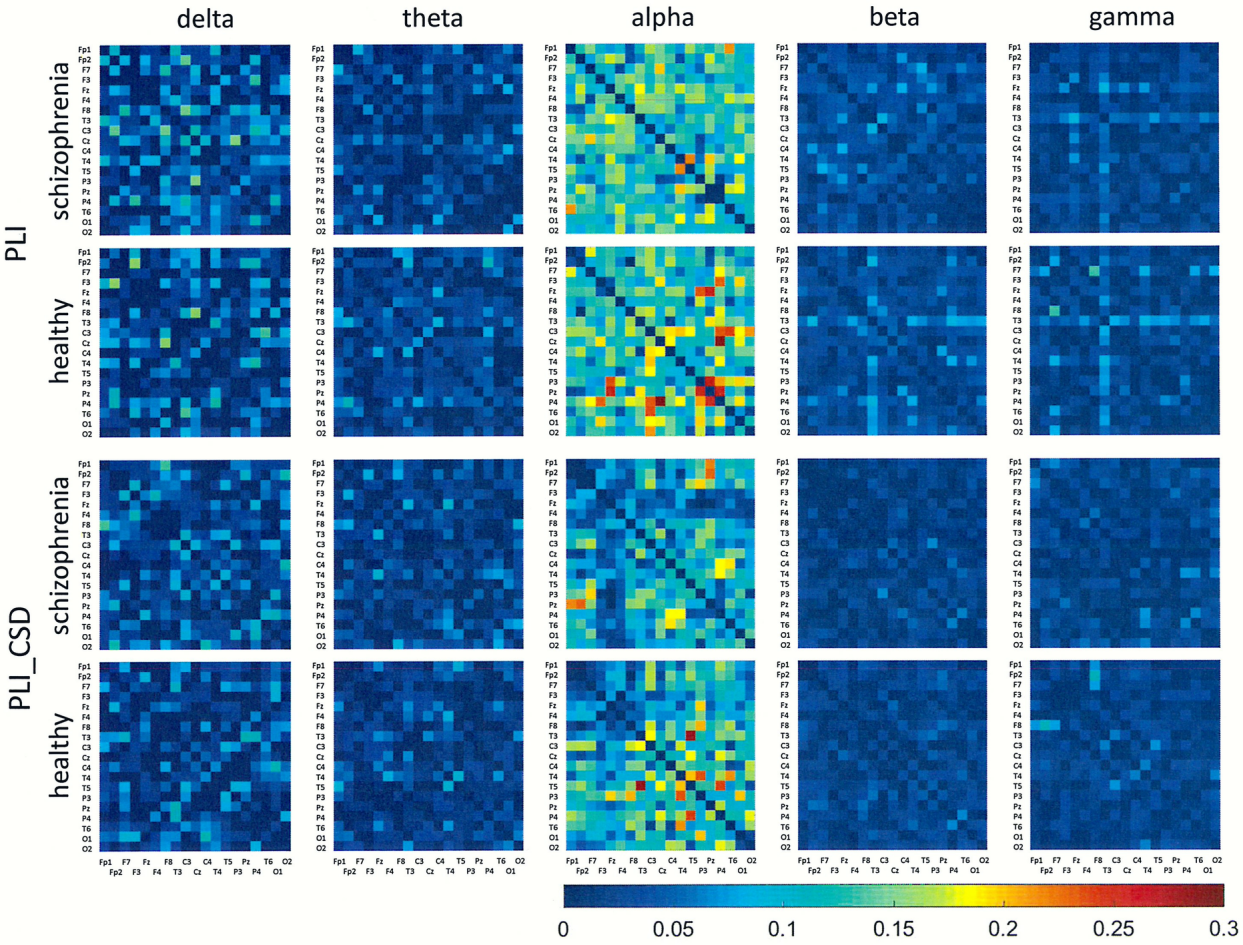
Mean adjacency matrices for PLI in patients with schizophrenia and healthy controls for delta, theta, alpha, beta, and gamma frequency bands using FCz or CSD as a reference.

**Fig 3:**
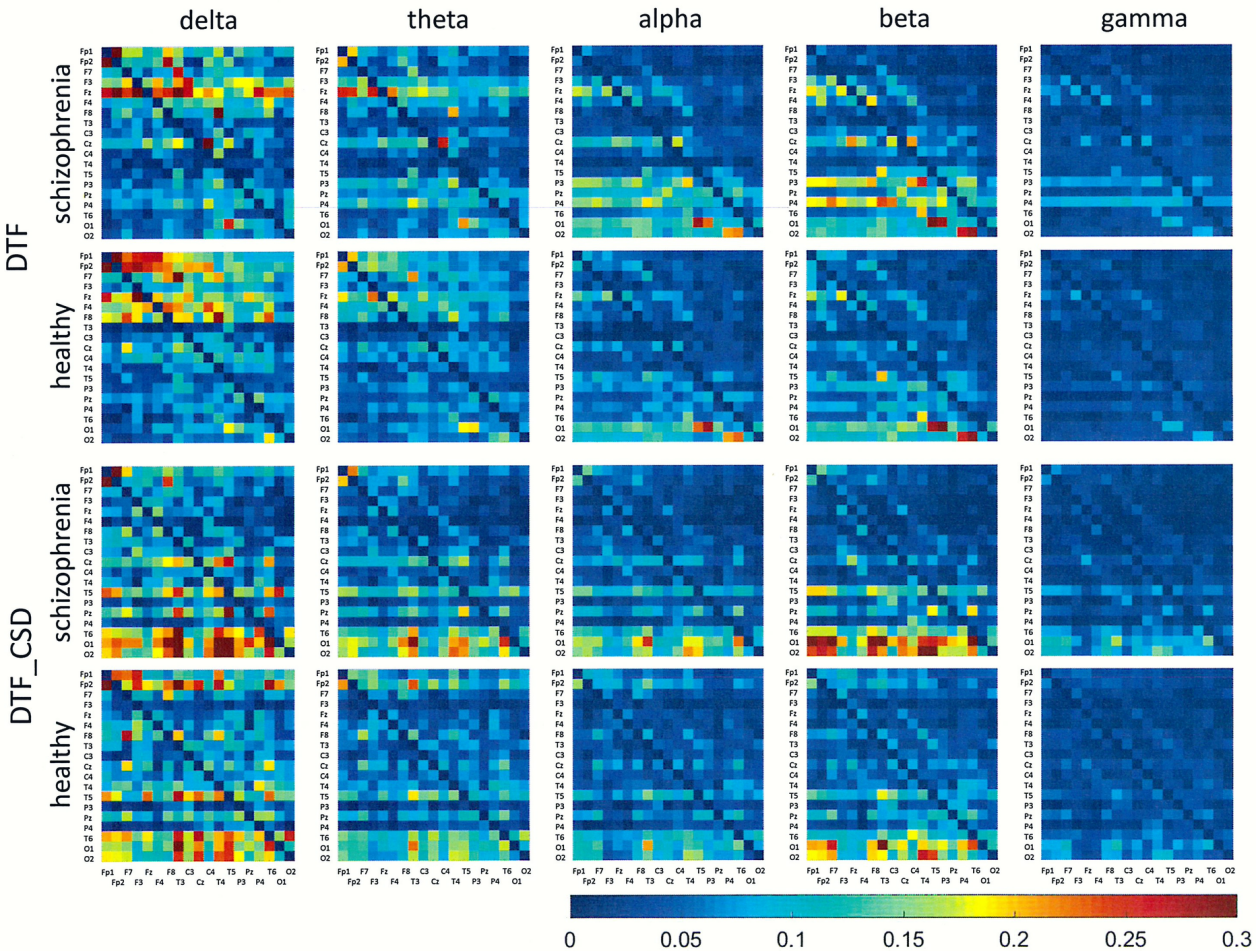
Mean adjacency matrices for DTF in patients with schizophrenia and healthy controls for delta, theta, alpha, beta, and gamma frequency bands using FCz or CSD as a reference. Information flows from the electrodes marked on the vertical axis to the electrodes marked on the horizontal axis.

**Fig 4:**
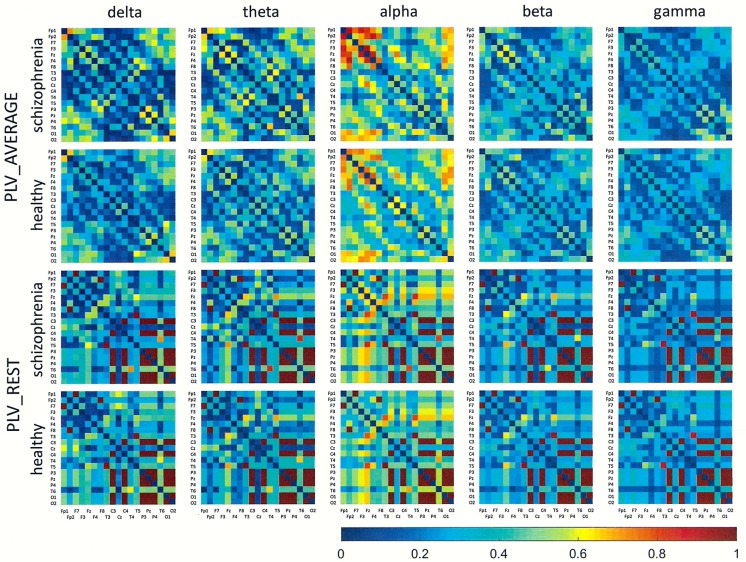
Mean adjacency matrices for PLV in patients with schizophrenia and healthy controls for delta, theta, alpha, beta, and gamma frequency bands following re-referencing using the average reference electrode (AVERAGE) or reference electrode standardization techniques (REST).

**Fig 5:**
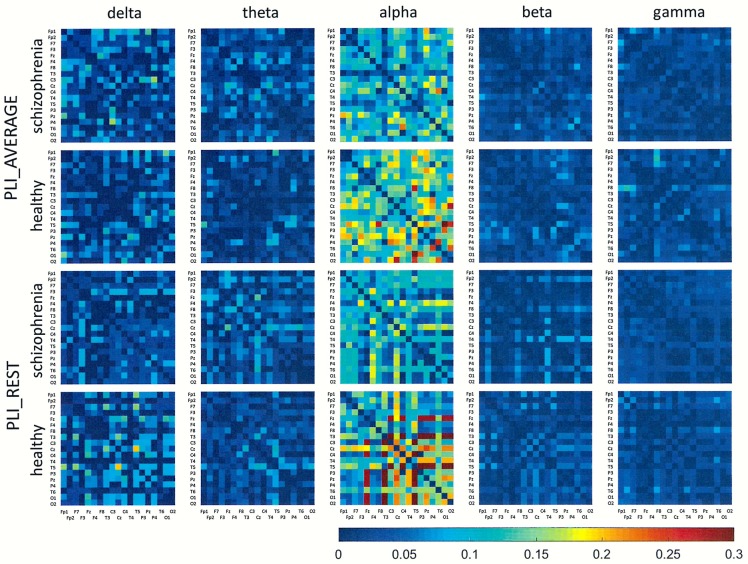
Mean adjacency matrices for PLI in patients with schizophrenia and healthy controls for delta, theta, alpha, beta, and gamma frequency bands following re-referencing using the average reference electrode (AVERAGE) or reference electrode standardization techniques (REST).

**Fig 6:**
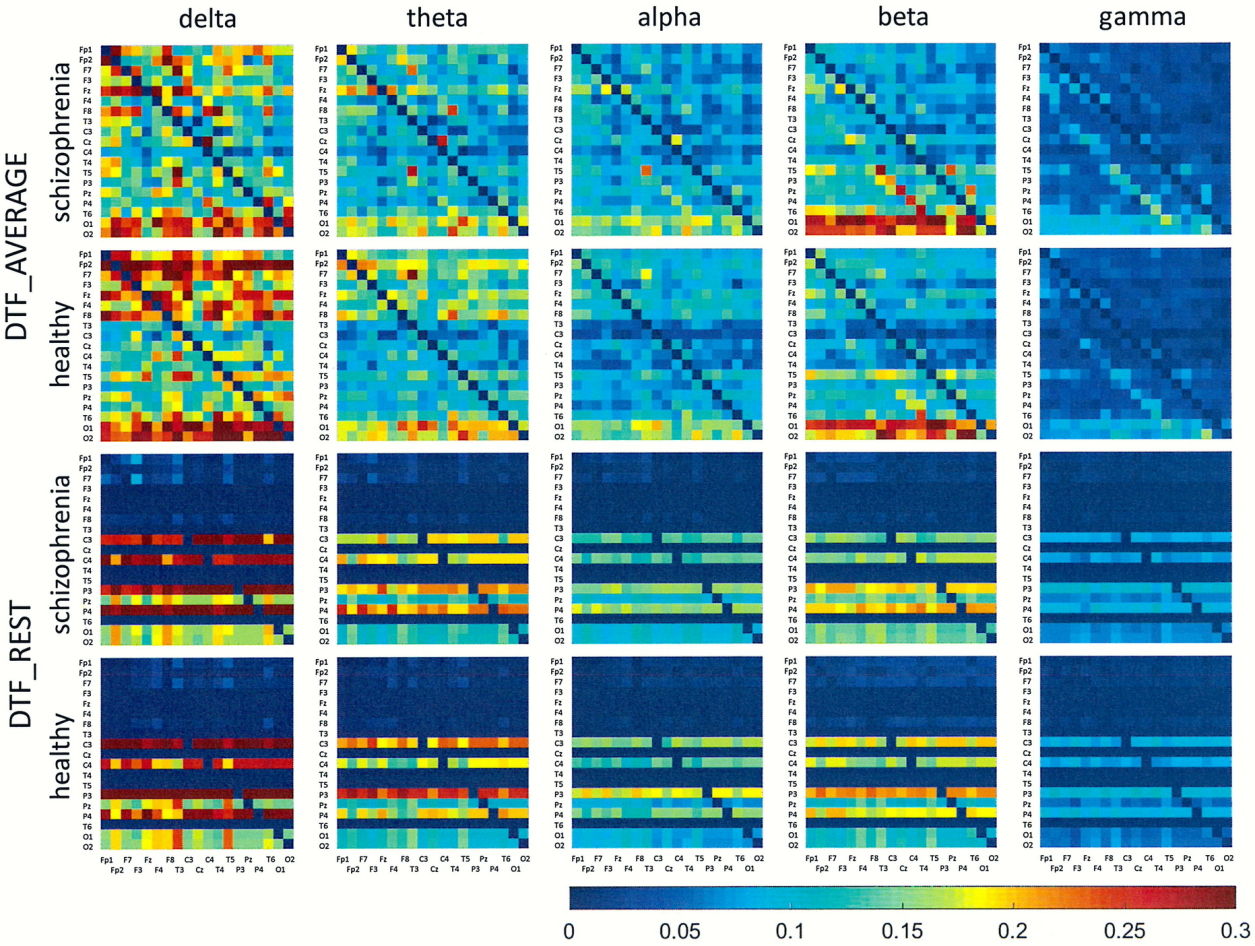
Mean adjacency matrices for DTF in patients with schizophrenia and healthy controls for delta, theta, alpha, beta, and gamma frequency bands following re-referencing using the average reference electrode (AVERAGE) or reference electrode standardization techniques (REST). Information flows from the electrodes marked on the vertical axis to the electrodes marked on the horizontal axis.

Figs 1–6 show the average adjacency matrices calculated for patients with schizophrenia and healthy controls for three connectivity measures (PLV, PLI, and DTF) using four reference electrodes (FCz, CSD, AVERAGE, and REST). The matrices are shown for delta, theta, alpha, beta and gamma bands separately. Only the statistically significant differences are shown.

The differences between the two groups (healthy controls and schizophrenia patients) for graph-based indices (density, strength, degree) are shown for each frequency band separately in Tables 1–5. The first value is from healthy controls, and second from schizophrenia patients. These indices were calculated using three connectivity measures (PLV, PLI, DTF) and three montages (CSD, AVERAGE and REST). The calculations have been carried out at a threshold equal to 70, i.e., only 30% of strongest connections were taken into consideration.

**Table 1:** Differences between schizophrenia patients and healthy controls for the delta band.

index	CSD			AVERAGE				REST	
	PLV	PLI	DTF	PLV	PLI	DTF	PLV	PLI	DTF
**Density**	6.60±0.94 vs. 3.38±0.63, F = 8.082, p = 0.009	3.88±0.55 vs. 4.64±0.58, F = 0.890, p = 0.354	3.74±0.49 vs. 2.67±0.39, F = 2.938, p = 0.09	17.96±1.75 vs. 17.00±1.56, F = 0.168, p = 0.686	4.39±0.83, vs. 4.68±0.69, F = 0.073, p = 0.789	4.16±0.62 vs. 2.59±0.49, F = 3.969, p = 0.057	18.13±1.41 vs. 16.21±0.80, F = 1.340, p = 0.248	6.93±2.01 vs. 2.72±0.93, F = 3.645, p = 0.067	11.11±0.73 vs. 9.96±0.73, F = 1.246, p = 0.275
**Degree**	2.38±0.19 vs. 1.22±0.12, F = 34.317, p<0.000001	1.40±0.12 vs. 1.67±0.12, F = 2.356, p = 0.125	1.35±0.11 vs. 0.96±0.07, F = 9.032, p = 0.003	6.47±0.32 vs. 6.12±0.29, F = 0.829, p = 0.363	1.58±0.16 vs. 1.68±0.15, F = 0.238, p = 0.626	1.50±0.13 vs. 0.93±0.08, F = 14.599, p = 0.0002	6.53±0.37 vs. 5.83±0.33, F = 9.001, p = 0.003	2.50±0.27 vs. 0.98±0.14, F = 24.804, p = 0.000001	4.00±0.27 vs. 3.59±0.25, F = 3.310, p = 0.069
**Strength**	1.45±0.12 vs. 0.75±0.08, F = 29.370, p<0.000001	0.66±0.06 vs. 0.78±0.06, F = 1.976, p = 0.160	0.70±0.06 vs. 0.49±0.04, F = 9.722, p = 0.002	4.22±0.23 vs. 3.96±0.19, F = 0.913, p = 0.340	0.80±0.08 vs. 0.86±0.08, F = 0.289, p = 0.591	0.92±0.08 vs. 0.57±0.05, F = 15.439, p = 0.0001	6.15±0.35 vs. 5.63±0.33, F = 8.863, p = 0.003	1.30±0.14 vs. 0.58±0.09, F = 17.599, p = 0.00003	3.19±0.22 vs. 2.94±0.21, F = 2.057, p = 0.152

**Table 2:** Differences between schizophrenia patients and healthy controls for the theta band.

index	CSD			AVERAGE				REST	
	PLV	PLI	DTF	PLV	PLI	DTF	PLV	PLI	DTF
**density**	5.39±0.94 vs. 4.14±0.63, F = 1.230, p = 0.278	5.05±0.62 vs. 4.64±0.66, F = 0.213, p = 0.648	3.68±0.61 vs. 2.99±0.35, F = 0.961, p = 0.336	15.91±1.52 vs. 19.09±0.72, F = 3.562, p = 0.070	4.47±0.75 vs. 5.05±0.81, F = 0.281, p = 0.601	4.16±0.63 vs. 3.09±0.63, F = 1.448, p = 0.240	15.54±0.54 vs. 15.41±0.13, F = 0.051, p = 0.824	6.31±1.01 vs. 6.06±1.68, F = 0.016, p = 0.899	11.40±0.86 vs. 10.13±0.73, F = 1.274, p = 0.269
**degree**	1.94±0.17 vs. 1.49±0.12, F = 5.834, p = 0.016	1.82±0.14 vs. 1.67±0.13, F = 0.637, p = 0.425	1.32±0.11 vs. 1.08±0.08, F = 3.629, p = 0.057	5.73±0.24 vs. 6.87±0.23, F = 14.831, p = 0.0001	1.61±0.15 vs. 1.82±0.16, F = 0.893, p = 0.345	1.50±0.13 vs. 1.11±0.09, F = 6.371, p = 0.012	5.59±0.33 vs. 5.55±0.31, F = 0.211, p = 0.646	2.27±0.20 vs. 2.18±0.22, F = 0.091, p = 0.763	4.11±0.28 vs. 3.65±0.26, F = 3.963, p = 0.047
**strength**	1.07±0.10 vs. 0.77±0.06, F = 8.232, p = 0.004	0.56±0.04 vs. 0.54±0.04, F = 0.192, p = 0.661	0.43±0.04 vs. 0.35±0.03, F = 3.056, p = 0.081	3.42±0.14 vs. 4.25±0.15, F = 20.570, p = 0.00001	0.55±0.05 vs. 0.64±0.06, F = 1.475, p = 0.225	0.58±0.05 vs. 0.42±0.03, F = 7.800, p = 0.005	5.45±0.33 vs. 5.43±0.31, F = 0.086, p = 0.770	0.76±0.07 vs. 0.80±0.08, F = 0.104, p = 0.748	2.15±0.15 vs. 1.97±0.14, F = 2.263, p = 0.133

**Table 3:** Differences between schizophrenia patients and healthy controls for the alpha band.

index	CSD			AVERAGE				REST	
	PLV	PLI	DTF	PLV	PLI	DTF	PLV	PLI	DTF
**density**	13.70±2.52 vs. 10.03±1.98, F = 1.315, p = 0.262	7.60±2.02 vs. 6.06±1.15, F = 0.442, p = 0.512	2.67±0.50 vs. 3.61±0.49, F = 1.780, p = 0.191	31.24±4.40 vs. 27.49±2.88, F = 0.511, p = 0.481	12.16±3.07 vs. 7.89±2.28, F = 1.238, p = 0.276	3.03±0.49 vs. 3.65±0.71, F = 0.529, p = 0.474	29.20±5.45 vs. 24.35±3.02, F = 0.604, p = 0.444	18.34±3.93 vs. 12.61±3.08, F = 1.312, p = 0.263	10.90±0.87 vs. 9.46±0.47, F = 2.125, p = 0.157
**degree**	4.93±0.35 vs. 3.61±0.30, F = 10.122, p = 0.002	2.74±0.26 vs. 2.18±0.20, F = 2.857, p = 0.092	0.96±0.09 vs. 1.30±0.11, F = 7.673, p = 0.006	11.25±0.54 vs. 9.89±0.47, F = 4.954, p = 0.026	4.38±0.37 vs. 2.84±0.29, F = 10.353, p = 0.001	1.09±0.10 vs. 1.32±0.10, F = 2.675, p = 0.103	10.51±0.60 vs. 8.77±0.46, F = 8.378, p = 0.004	6.60±0.46 vs. 4.54±0.38, F = 11.804, p = 0.0006	3.92±0.26 vs. 3.41±0.24, F = 5.094, p = 0.024
**strength**	3.48±0.27 vs. 2.59±0.23, F = 7.524, p = 0.006	1.59±0.17 vs. 1.13±0.12, F = 4.551, p = 0.033	0.23±0.02 vs. 0.31±0.03, F = 8.251, p = 0.004	8.40±0.46 vs. 7.49±0.39, F = 3.043, p = 0.082	2.67±0.27 vs. 1.56±0.19, F = 11.074, p = 0.0009	0.33±0.03 vs. 0.36±0.03, F = 0.560, p = 0.455	9.39±0.52 vs. 7.92±0.41, F = 9.431, p = 0.002	3.75±0.31 vs. 2.33±0.23, F = 13.530, p = 0.0003	1.54±0.10 vs. 1.37±0.10, F = 3.768, p = 0.053

**Table 4:** Differences between schizophrenia patients and healthy controls for the beta band.

index	CSD			AVERAGE				REST	
	PLV	PLI	DTF	PLV	PLI	DTF	PLV	PLI	DTF
**density**	4.72±0.91 vs. 3.17±0.40, F = 2.406, p = 0.133	4.05±0.80 vs. 4.39±0.74, F = 0.094, p = 0.761	2.61±0.40 vs. 3.24±0.32, F = 1.477, p = 0.235	14.45±1.69 vs. 13.07±1.31, F = 0.415, p = 0.525	4.72±0.78 vs. 6.14±0.95, F = 1.338, p = 0.258	2.94±0.37 vs. 3.03±0.39, F = 0.024, p = 0.878	14.24±0.10 vs. 14.75±0.15, F = 7.610, p = 0.010	8.27±1.87 vs. 8.15±1.88, F = 0.002, p = 0.963	10.53±1.07 vs. 10.40±0.66, F = 0.010, p = 0.922
**degree**	1.70±0.15 vs. 1.14±0.10, F = 11.168, p = 0.0009	1.46±0.13 vs. 1.58±0.12, F = 0.453, p = 0.501	0.94±0.08 vs. 1.17±0.11, F = 4.427, p = 0.036	5.20±0.26 vs. 4.71±0.24, F = 2.762, p = 0.097	1.70±0.16 vs. 2.21±0.18, F = 4.740, p = 0.030	1.06±0.09 vs. 1.09±0.10, F = 0.070, p = 0.792	5.13±0.33 vs. 5.31±0.32, F = 19.102, p = 0.00002	2.98±0.27 vs. 2.93±0.29, F = 0.014, p = 0.907	3.79±0.27 vs. 3.74±0.24, F = 0.036, p = 0.849
**strength**	0.80±0.07 vs. 0.56±0.05, F = 8.884, p = 0.003	0.27±0.02 vs. 0.26±0.02, F = 0.183, p = 0.669	0.30±0.03 vs. 0.41±0.04, F = 8.865, p = 0.003	2.80±0.14 vs. 2.68±0.14, F = 0.479, p = 0.489	0.36±0.04 vs. 0.41±0.04, F = 1.215, p = 0.271	0.42±0.04 vs. 0.46±0.04, F = 0.597, p = 0.440	5.09±0.33 vs. 5.23±0.32, F = 19.954, p = 0.00001	0.59±0.06 vs. 0.51±0.05, F = 0.999, p = 0.318	1.81±0.13 vs. 1.70±0.11, F = 0.897, p = 0.344

**Table 5:** Differences between schizophrenia patients and healthy controls for the gamma band.

index	CSD			AVERAGE				REST	
	PLV	PLI	DTF	PLV	PLI	DTF	PLV	PLI	DTF
**density**	4.43±0.84 vs. 3.59±0.62, F = 0.644, p = 0.430	2.21±0.43 vs. 4.55±0.46, F = 13.820, p = 0.0009	2.53±0.38 vs. 3.13±0.45, F = 1.055, p = 0.314	12.87±1.61 vs. 11.61±1.65, F = 0.295, p = 0.591	3.51±0.84 vs. 4.01±0.71, F = 0.210, p = 0.651	2.09±0.30 vs. 2.44±0.42, F = 0.478, p = 0.495	14.08±0.10 vs. 14.70±0.29, F = 4.295, p = 0.048	8.69±1.85 vs. 8.40±1.74, F = 0.013, p = 0.909	10.09±0.62 vs. 8.48±0.69, F = 3.000, p = 0.095
**degree**	1.59±0.15 vs. 1.29±0.12, F = 2.644, p = 0.105	0.80±0.10 vs. 1.64±0.12, F = 28.321, p<0.000001	0.91±0.08 vs. 1.13±0.10, F = 3.885, p = 0.049	4.63±0.27 vs. 4.18±0.25, F = 2.017, p = 0.156	1.26±0.14 vs. 1.44±0.13, F = 0.841, p = 0.360	0.75±0.07 vs. 0.88±0.08, F = 1.914, p = 0.167	5.07±0.33 vs. 5.29±0.33, F = 10.695, p = 0.001	3.13±0.29 vs. 3.02±0.28, F = 0.067, p = 0.795	3.63±0.24 vs. 3.05±0.22, F = 8.891, p = 0.003
**strength**	0.81±0.08 vs. 0.71±0.07, F = 0.872, p = 0.351	0.20±0.02 vs. 0.33±0.03, F = 13.400, p = 0.0003	0.12±0.01 vs. 0.18±0.02, F = 10.208, p = 0.001	2.37±0.14 vs. 2.18±0.13, F = 1.340, p = 0.248	0.30±0.03 vs. 0.31±0.03, F = 0.172, p = 0.678	0.14±0.01 vs. 0.17±0.02, F = 2.244, p = 0.135	5.04±0.33 vs. 5.21±0.33, F = 10.899, p = 0.001	0.67±0.06 vs. 0.64±0.06, F = 0.085, p = 0.770	0.86±0.06 vs. 0.73±0.05, F = 8.040, p = 0.005

Statistically significant differences between schizophrenia patients and healthy controls were observed for connectivity strength at the alpha band for each connectivity measure using the CSD transform or the REST re-referencing procedure (c.f. Table 3). Reduced synchronization of alpha waves was observed in patients with schizophrenia, compared to the healthy controls, and this pattern extended across the entire brain (PLV values greater than 0.3 and PLI values greater than 0.12 in Figs 1 and 2 and Figs 4 and 5 for CSD and REST, respectively). This reduced synchronization was accompanied by an increased flow of information, quantified by the strength of DTF connectivity when the CSD transform was used (c.f. Table 3). When the AVERAGE was used, decreased synchronization was observed for the PLI alone.

Group differences for other bands were also observed. The strength of PLV decreased in all frequency bands, except gamma band, when the CSD transform was used. The REST re-referencing procedure revealed an additional increase of PLV strength in higher frequency bands (beta and gamma). PLI is a more selective measure. The strength of PLI decreased in the alpha band for all reference electrodes. Moreover, this index decreased in the delta band when REST was applied and increased in gamma band when CSD was applied. The strength of DTF decreased for lower frequency bands: delta (for CSD and AVERAGE), theta (for AVERAGE) and alpha (for REST), and increased for higher frequency bands: alpha (for CSD), beta (for CSD) and gamma (for CSD and REST).

**Fig 7:**
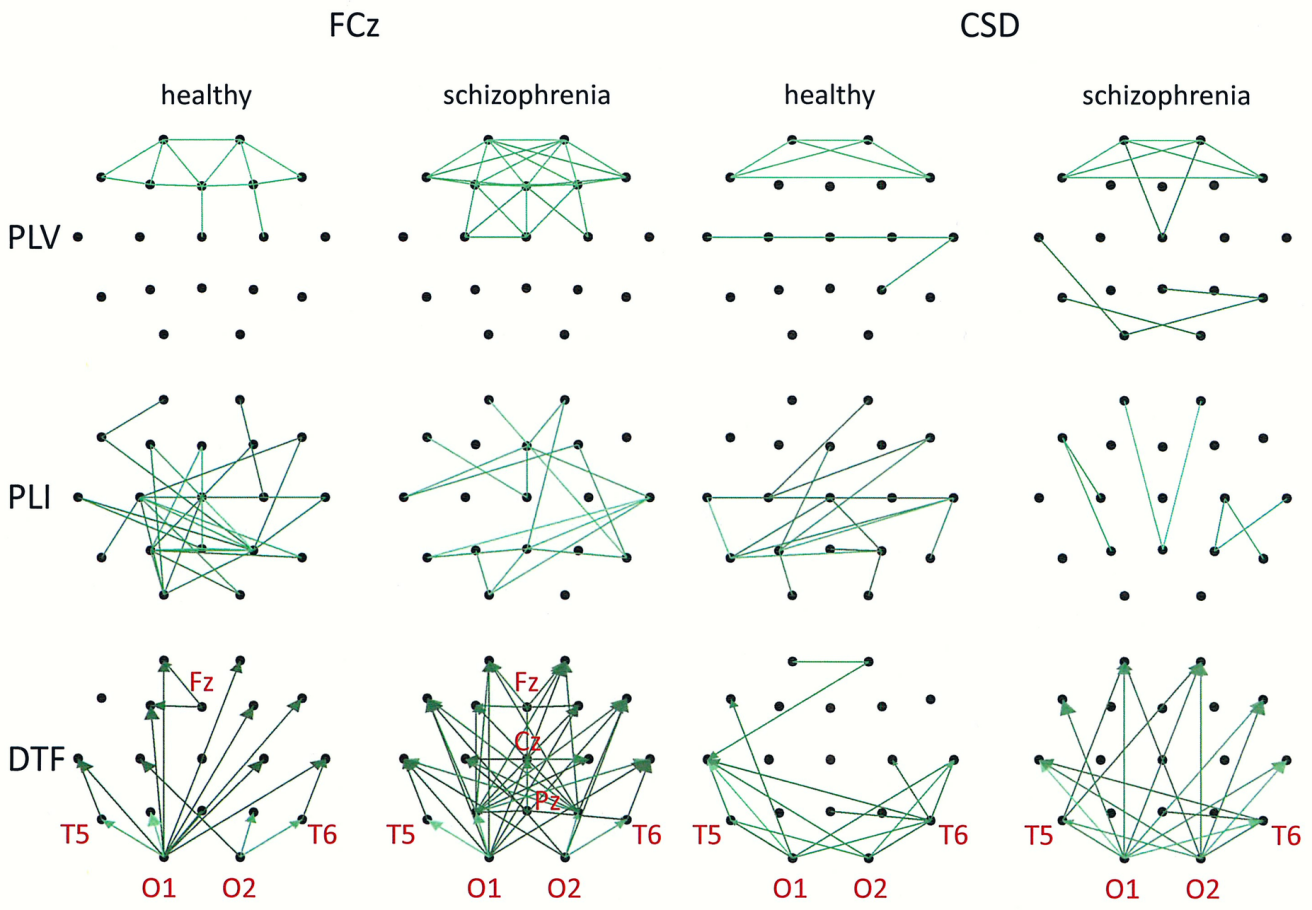
Group differences for the alpha band illustrated in the form of graphs for three connectivity measures (PLV, PLI, DTF) and two montages (FCz and CSD). Only the strongest connections are shown (PLV: 30% for FCz, 50% for CSD; PLI: 82% for FCz and for CSD; DTF: 52% for FCz, 40% for CSD). Directions of information flow, determined by DTF, are indicated using arrows.

**Fig 8:**
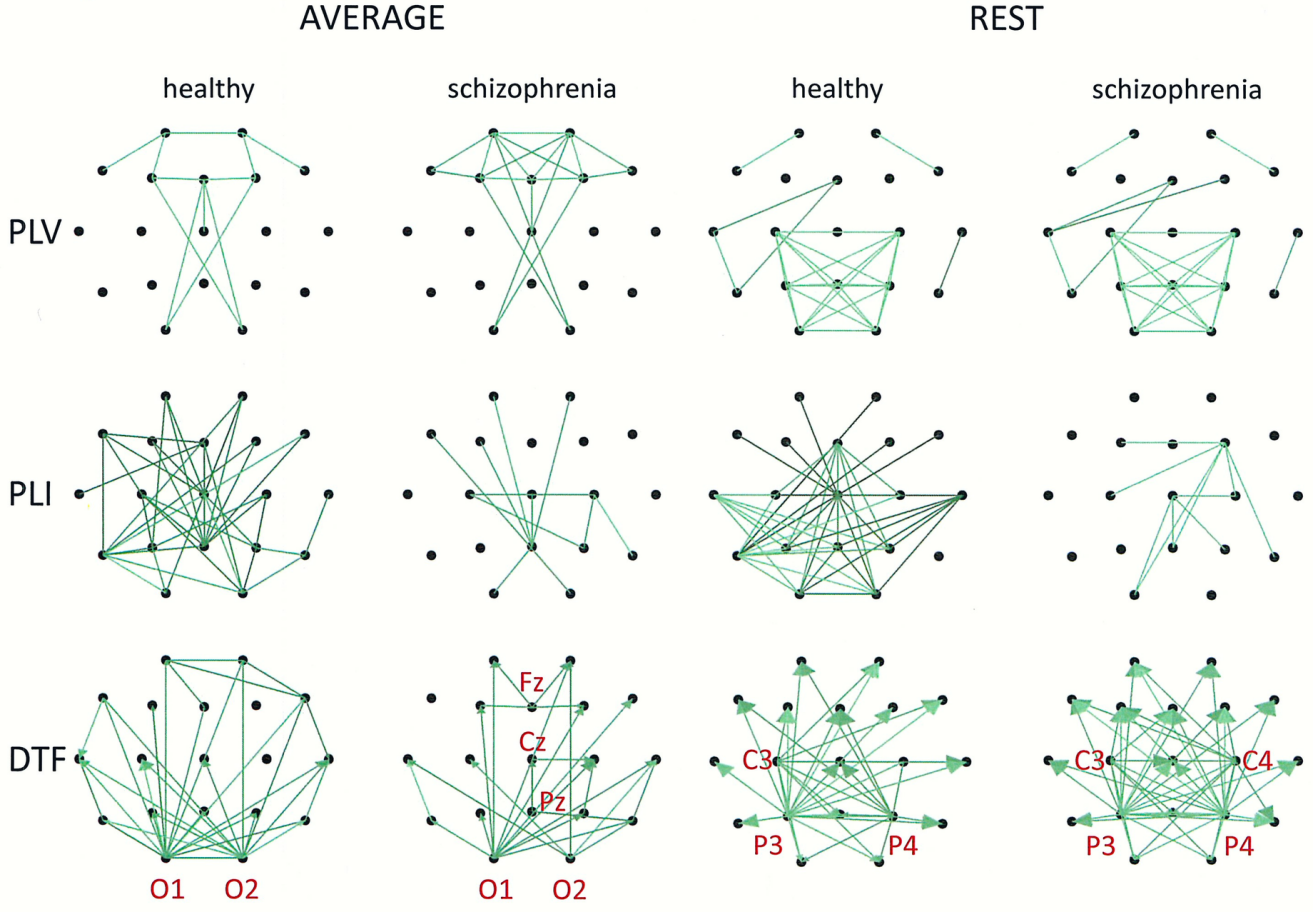
Group differences for the alpha band illustrated in the form of graphs for three connectivity measures (PLV, PLI, DTF) and two montages (AVERAGE and REST). Only the strongest connections are shown (PLV, DTF: 30% for AVERAGE and for REST; PLI: 82% for AVERAGE and for REST). Directions of information flow, determined by DTF, are indicated using arrows.

Group differences in connectivity for the alpha band are illustrated in Figs 7 and 8. Directional measures, such as DTF, allow for identification of the main sources and directions of information flow. Directions of information flow, determined by DTF, are indicated by the arrows. Only the strongest connections are shown. In patients with schizophrenia, there is a greater number of flows from the occipital to the frontal part of the brain, corresponding to weaker PLI which is independent of the reference electrode. Moreover, the target of information flow, in particular the frontal region, was an area of greater PLV when FCz, CSD or AVERAGE were applied. Thus, the area and the strength of synchronization depends on the connectivity measure–in patients with schizophrenia, more synchronized frontal brain regions, quantified by PLV, corresponds to less synchronized posterior brain regions, quantified by PLI. An exception is the REST, for which the area of synchronization quantified by PLV was located to posterior brain regions. Another difference between REST and other reference electrodes consisted of source localization. The main sources are located in the occipital lobe (electrodes O1 and O2) for each electrode reference, excluding REST where they are located mainly in the parietal (P3 and P4) and central (C3 and C4) part of brain (see adjacency matrices in Fig 6 and graphs for DTF in Fig 8).

Comparing the results for FCz with the results for other reference electrodes, revealed a decrease in strength at Fz and Cz, where the reference electrode was placed, when CSD or REST was used (c.f. rows in the connectivity matrices in Figs 3 and 6, and graphs for DTF in Figs 7 and 8). The differences between the adjacency matrices for FCz and CSD or REST were present particularly for the bivariate measures such as PLV and PLI (c.f. Figs 1 and 2 for CSD and Figs 4 and 5 for REST). CSD as well as REST transforms allow for the elimination of false indirect connections related to the impact of common sources (volume conduction). The reduction of connectivity in the alpha band can be observed in the adjacency matrices of PLV for frontal areas of brain (the upper left corner of the adjacency matrix with PLV greater than 0.8) and the connectivity between neighbouring electrodes (lines with higher PLV values parallel to diagonals visible in every band). For PLI adjacency matrices, the connections in the frontal part of brain (the upper left corner of adjacency matrices) were eliminated.

**Fig 9:**
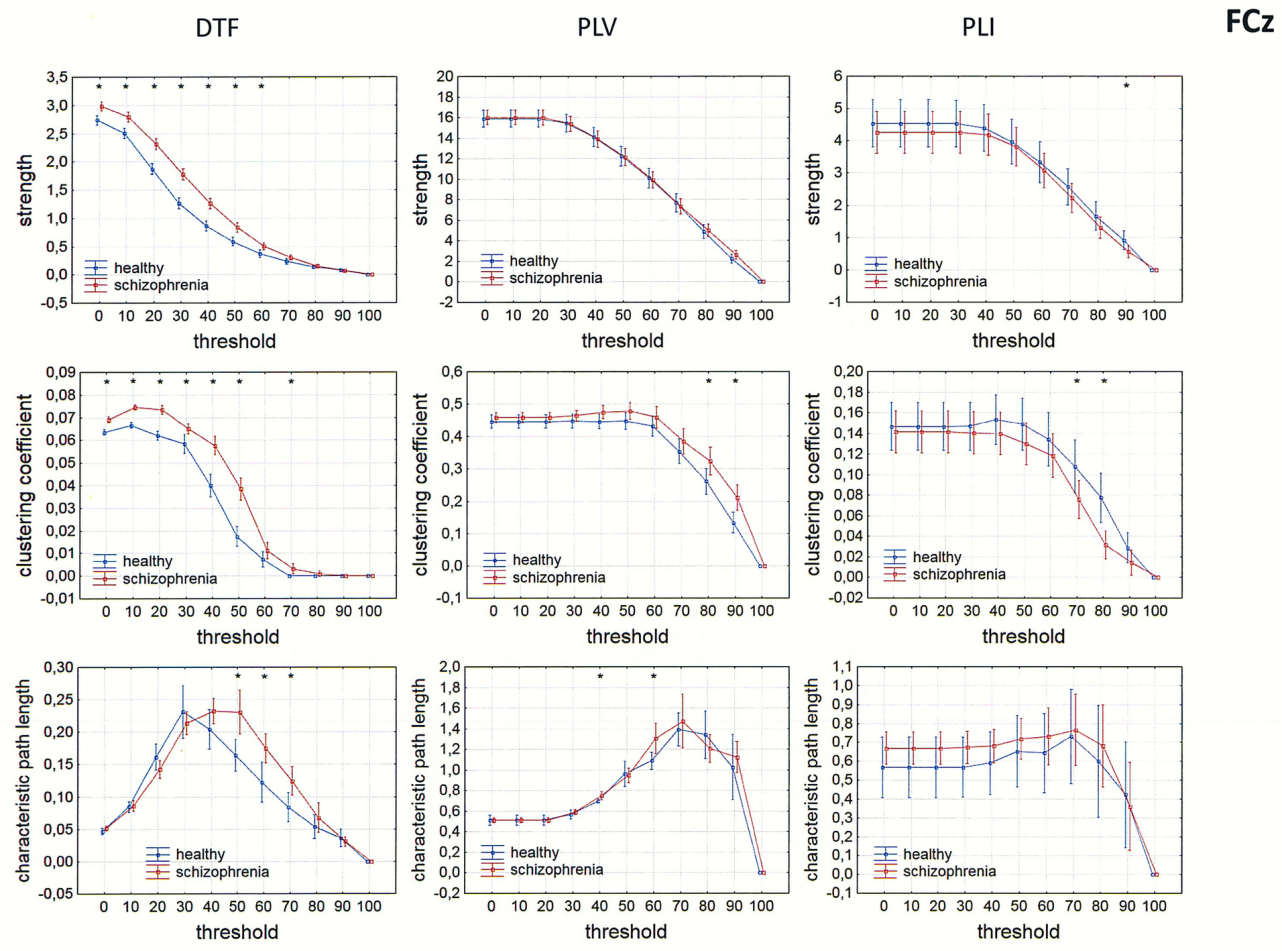
Comparison of strength, clustering coefficient, and characteristic path length in patients with schizophrenia and healthy controls for three connectivity measures (DTF, PLV and PLI) in the alpha band for FCz. The sum of inward and outward links was considered in the case of the index strength for DTF. The asterisks at the top of each chart indicate significant differences between the patients with schizophrenia and controls.

**Fig 10:**
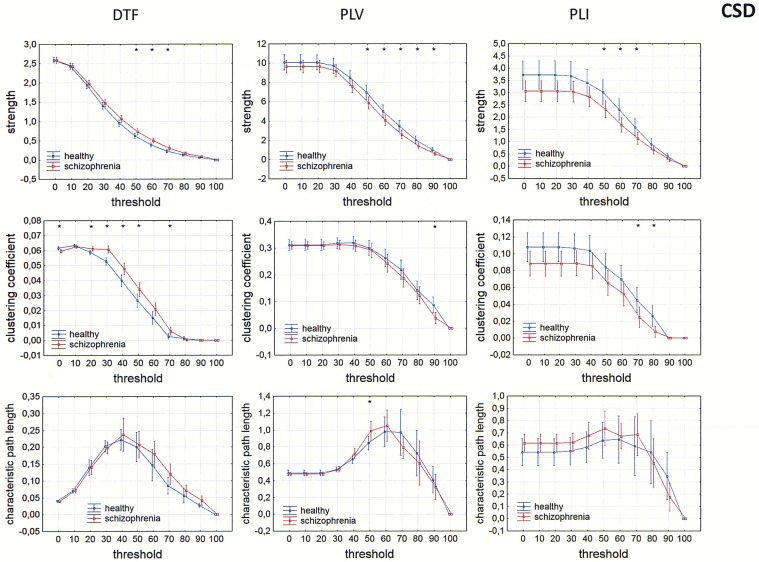
Comparison of strength, clustering coefficient, and characteristic path length in patients with schizophrenia and healthy controls for three connectivity measures (DTF, PLV and PLI) in the alpha band for CSD. The sum of inward and outward links was considered in the case of the index strength for DTF. The asterisks at the top of each chart indicate significant differences between the patients with schizophrenia and controls.

**Fig 11:**
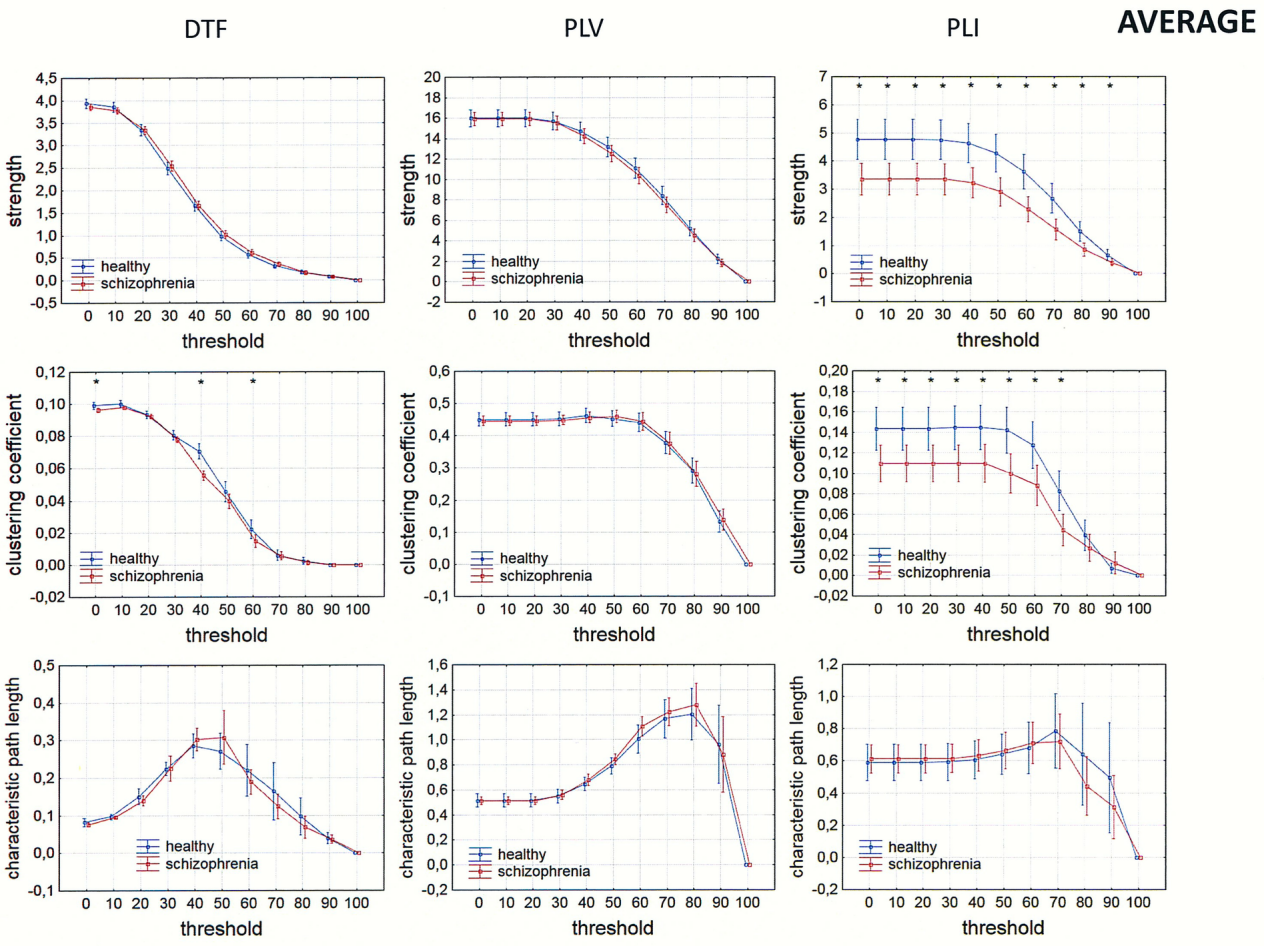
Comparison of strength, clustering coefficient, and characteristic path length in patients with schizophrenia and healthy controls for three connectivity measures (DTF, PLV and PLI) in the alpha band for AVERAGE. The sum of inward and outward links was considered in the case of the index strength for DTF. The asterisks at the top of each chart indicate significant differences between the patients with schizophrenia and controls.

**Fig 12:**
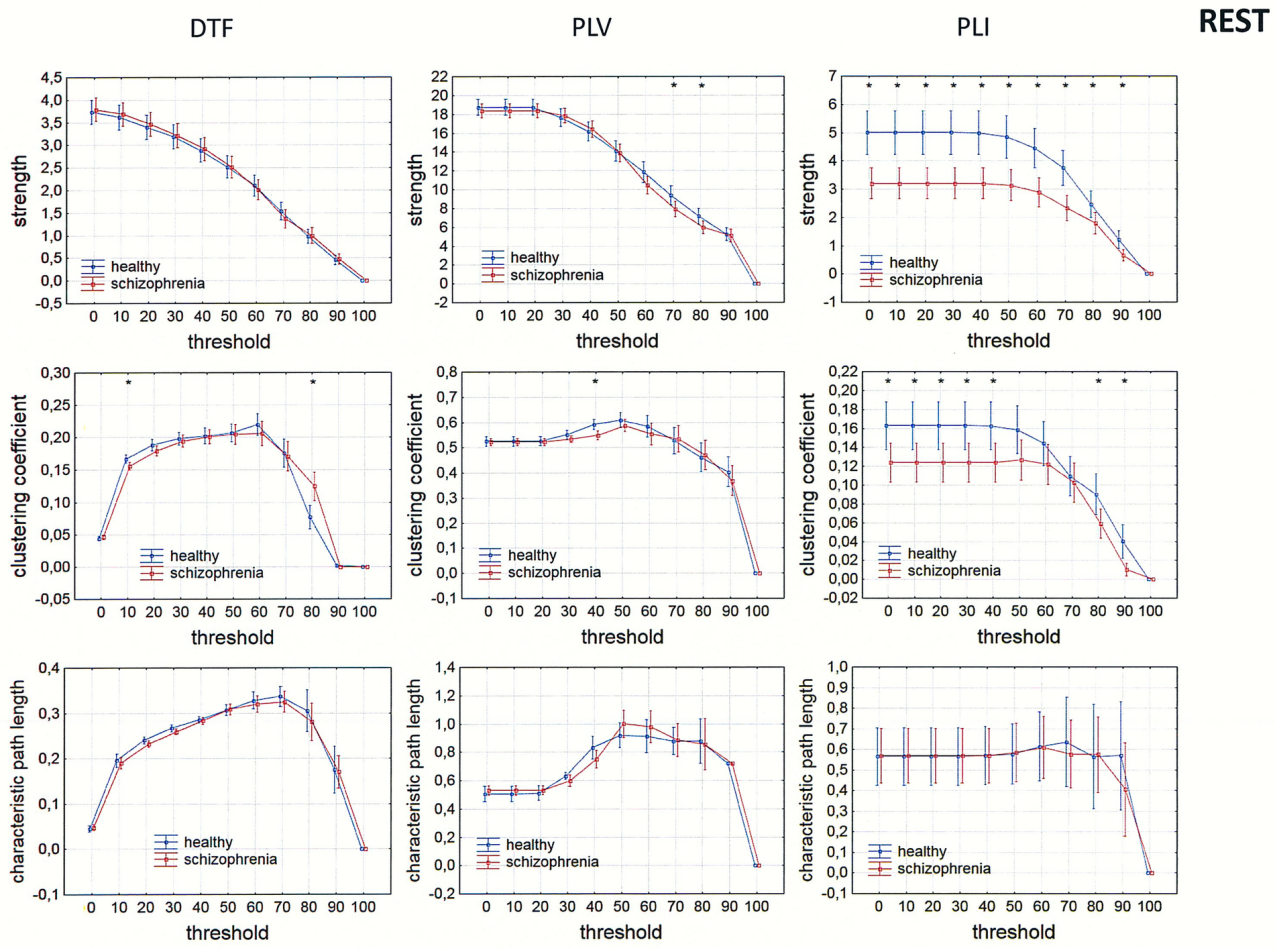
Comparison of strength, clustering coefficient, and characteristic path length in patients with schizophrenia and healthy controls for three connectivity measures (DTF, PLV and PLI) in the alpha band for REST. The sum of inward and outward links was considered in the case of the index strength for DTF. The asterisks at the top of each chart indicate significant differences between the patients with schizophrenia and controls.

Differences between the groups were also observed in measures of integration (characteristic path length) and segregation (clustering coefficient). Comparison of strength, clustering coefficient, and characteristic path length in both groups is presented in Figs 9–12 for the alpha band, as a function of threshold separately for each of reference electrode. A threshold of zero means that all statistically significant connections were considered. Increasing the threshold by 10 means that 10% of the weakest connections were eliminated. The network is highly connected and the elimination of even 90% of the weakest connections does not completely destroy the characteristic connectivity pattern. Statistically significant differences between the groups were observed over a wide range of thresholds. Lower clustering coefficient and a shorter characteristic path length in the alpha band were found in the patients with schizophrenia when compared to healthy persons for both measures of phase synchronization (PLV and PLI) when CSD, AVERAGE or REST were applied. However, for the clustering coefficient the group differences were bigger and they were in the wider range of threshold for PLI than for PLV, particularly when AVERAGE or REST was applied. An opposite relation was found for the clustering coefficient using the DTF when FCz, CSD or REST was applied.

**Fig 13:**
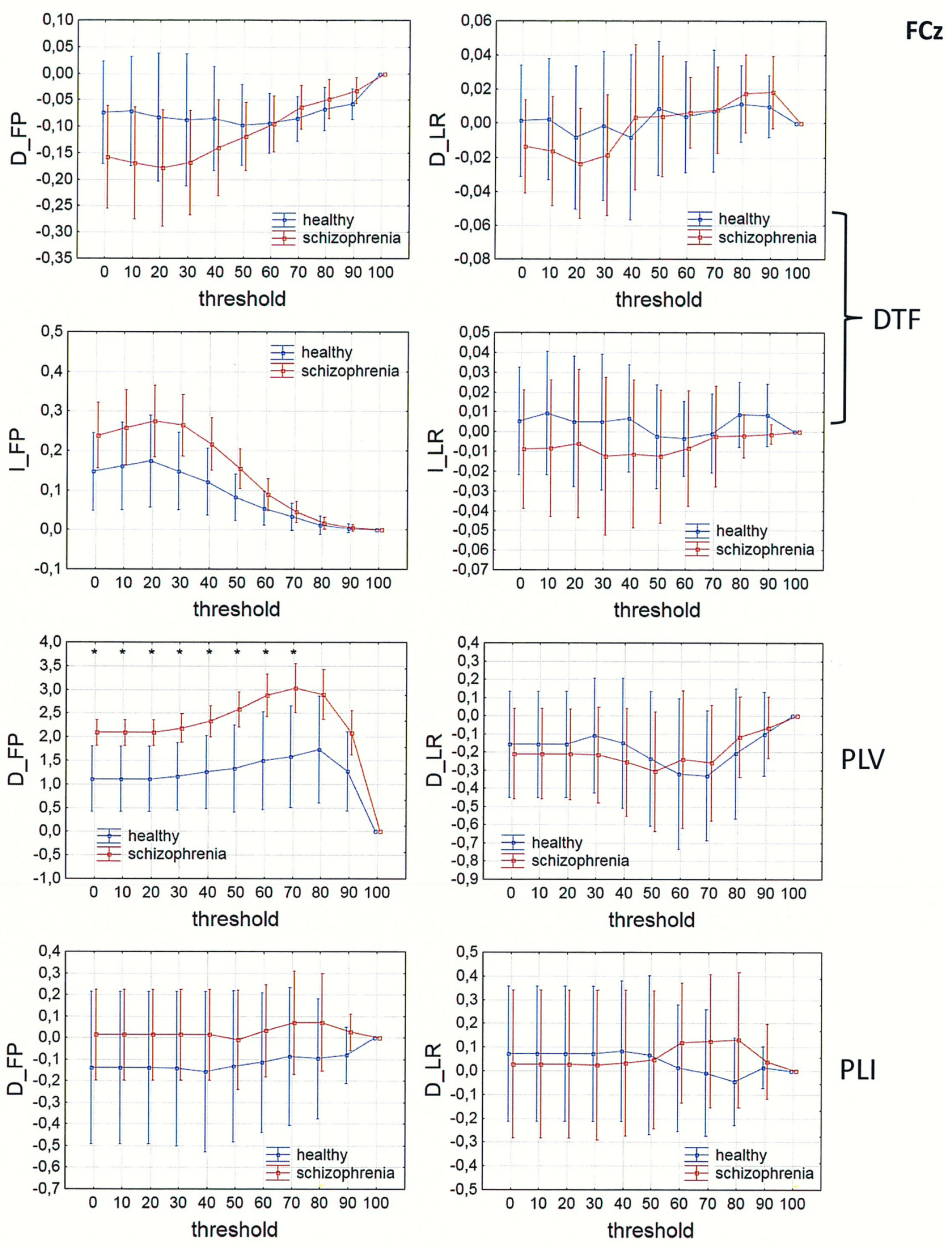
Comparison of asymmetry measures: D_FP_, D_LR_, I_FP_ and I_LR_ for the alpha band in patients with schizophrenia and healthy controls as a function of threshold for three connectivity measures (DTF, PLV and PLI) calculated for FCz. The asterisks at the top of each chart indicate significant differences between the patients with schizophrenia and controls.

**Fig 14:**
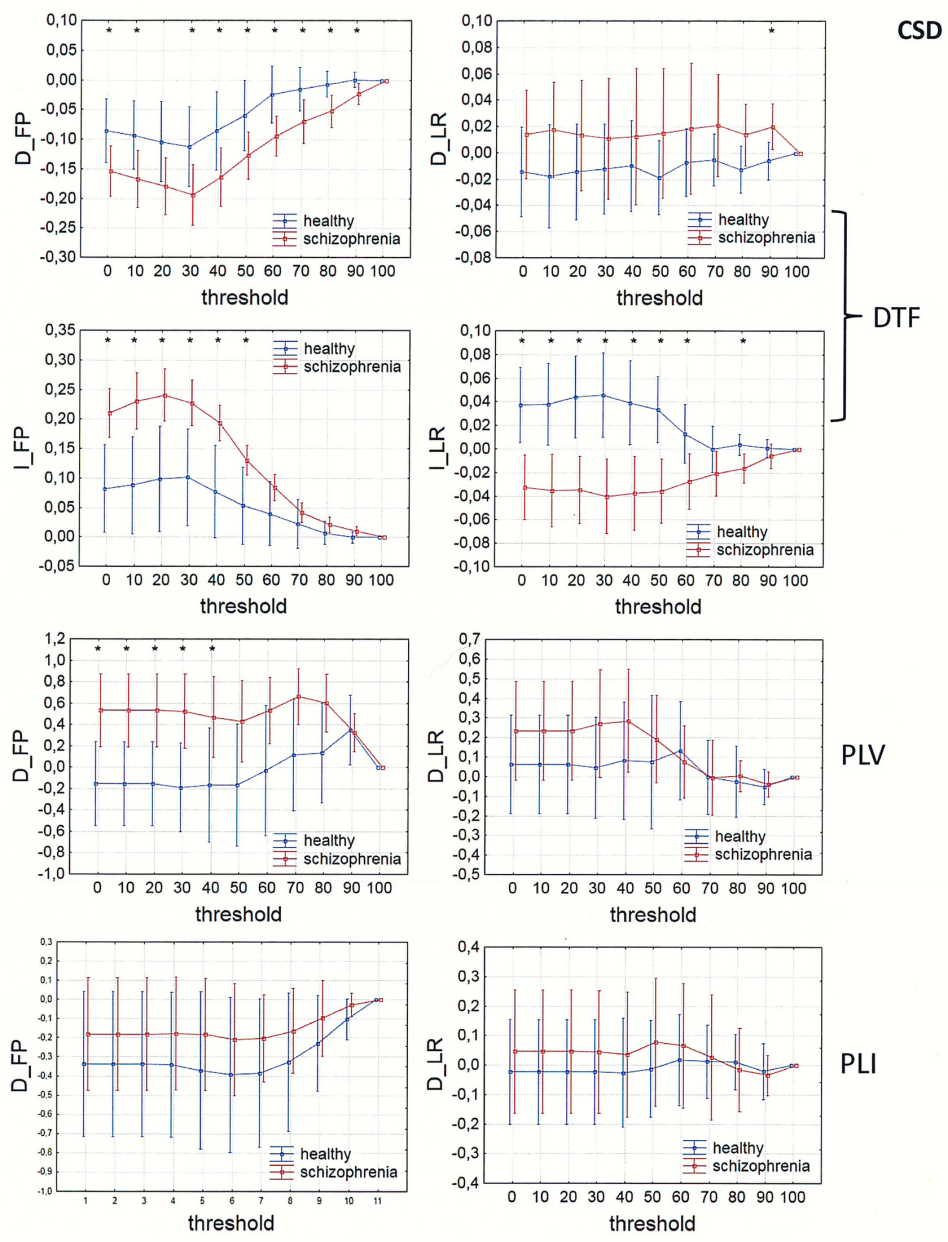
Comparison of asymmetry measures: D_FP_, D_LR_, I_FP_ and I_LR_ for the alpha band in patients with schizophrenia and healthy controls as a function of threshold for three connectivity measures (DTF, PLV and PLI) calculated for CSD. The asterisks at the top of each chart indicate significant differences between the patients with schizophrenia and controls.

**Fig 15:**
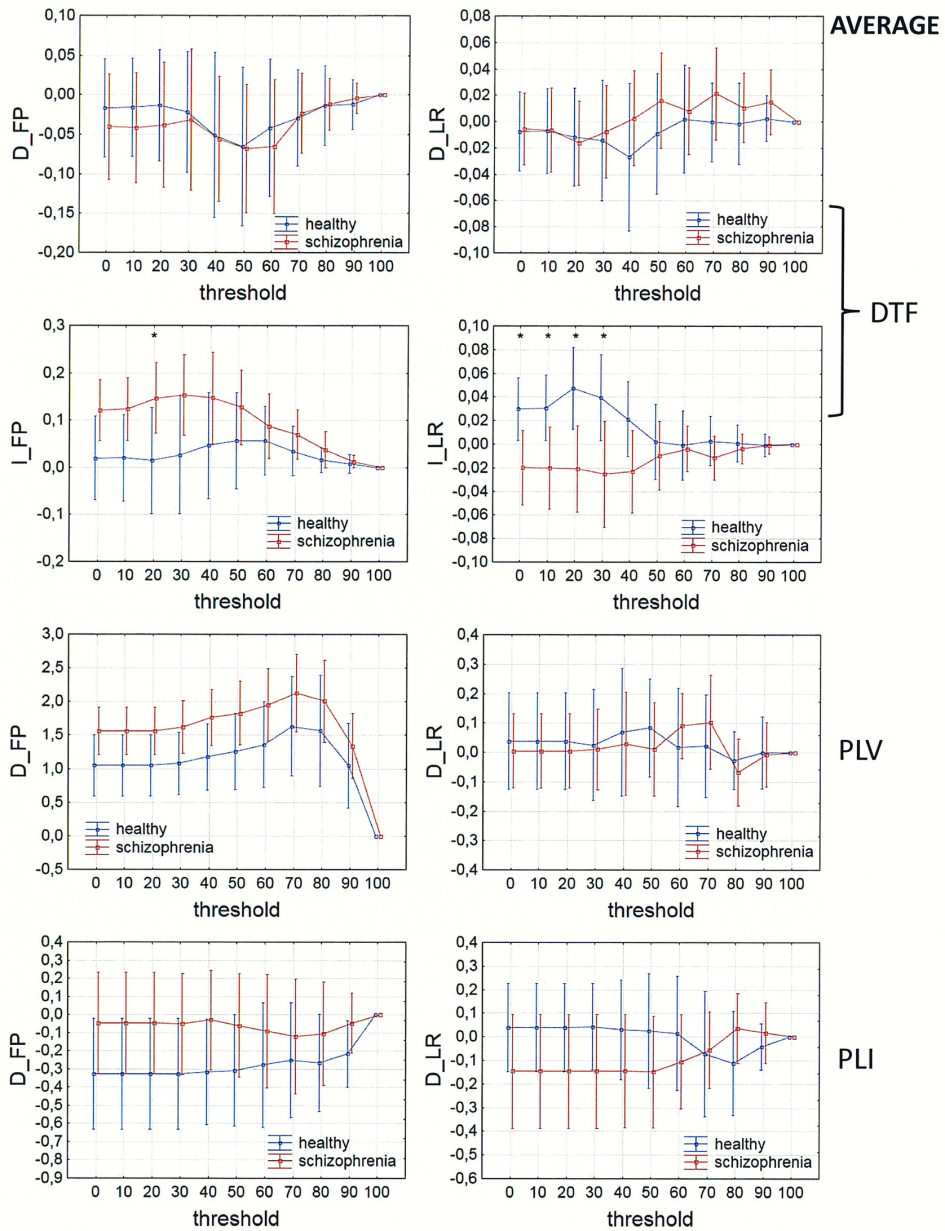
Comparison of asymmetry measures: D_FP_, D_LR_, I_FP_ and I_LR_ for the alpha band in patients with schizophrenia and healthy controls as a function of threshold for three connectivity measures (DTF, PLV and PLI) calculated for AVERAGE. The asterisks at the top of each chart indicate significant differences between the patients with schizophrenia and controls.

**Fig 16:**
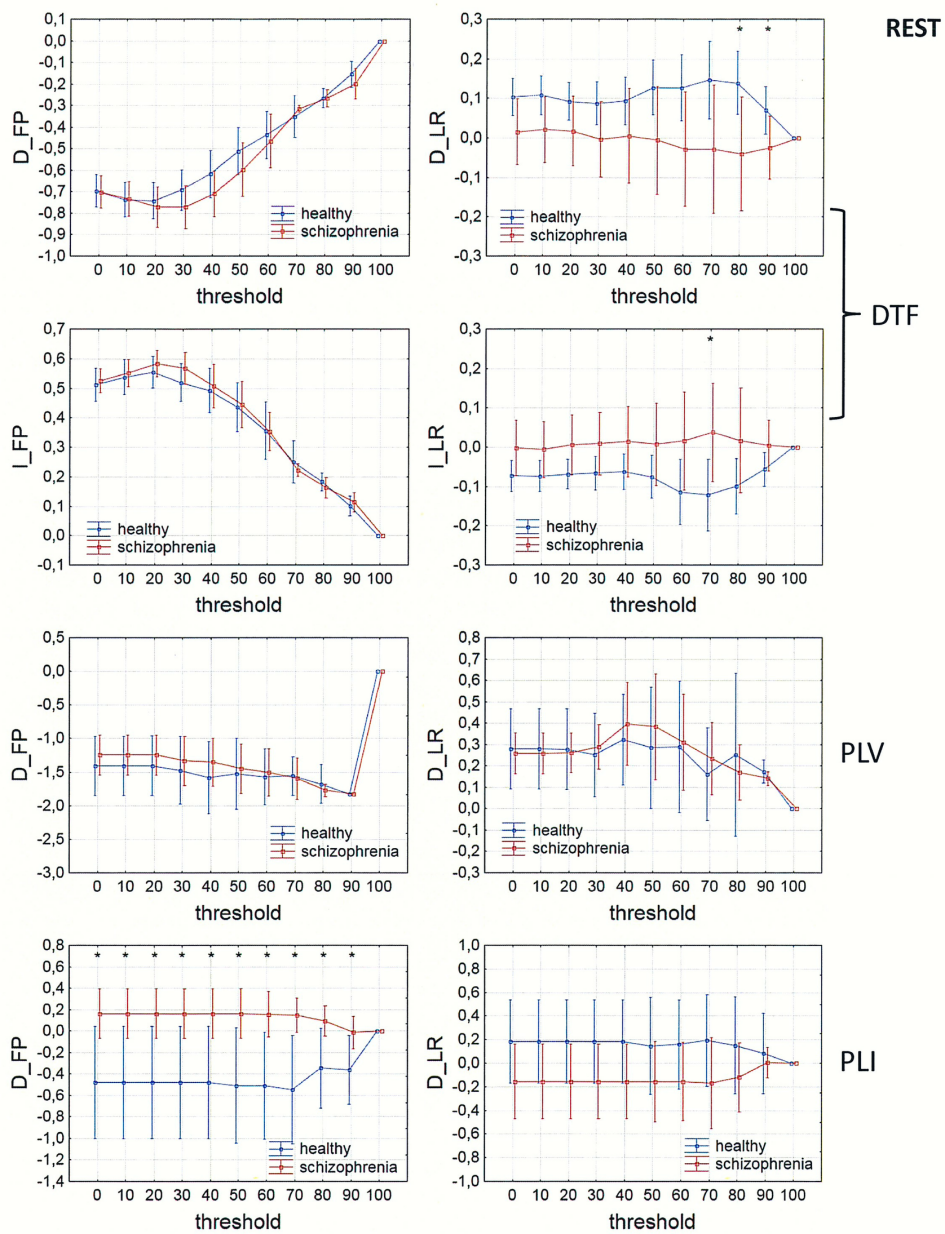
Comparison of asymmetry measures: D_FP_, D_LR_, I_FP_ and I_LR_ for the alpha band in patients with schizophrenia and healthy controls as a function of threshold for three connectivity measures (DTF, PLV and PLI) calculated for REST. The asterisks at the top of each chart indicate significant differences between the patients with schizophrenia and controls.

In Figs 13–16, a comparison of the asymmetry measures (D_FP_, D_LR_, I_FP_ and I_LR_) for both groups is shown for three connectivity measures in the alpha band as a function of threshold. For phase synchronization measures (PLV and PLI) only two asymmetry indices (D_FP_ and D_LR_) were evaluated, because the other two indices (I_FP_ and I_LR_) take zero values for symmetric matrices.

The results of PLV and DTF demonstrated group differences in fronto-posterior asymmetry using CSD transformed data (in range of threshold from 0 to 90 for DTF and from 0 to 40 for PLV), while for PLI the differences were significant only when REST was used (in range of threshold from 0 to 90). Synchronization, quantified by PLV as well as by PLI, was higher in patients with schizophrenia in comparison to healthy controls. This was accompanied by reduced information flows within the frontal part of the brain (lower index D_FP_ for DTF), but increased flows from frontal to posterior parts of the brain (higher index I_FP_ for DTF in range of threshold from 0 to 50).

Inter-hemispheric asymmetry revealed few differences between the groups. The only measure that revealed group differences was DTF, calculated for REST, using a narrow threshold range (threshold = 80–90 for D_LR_, and threshold = 70 for I_LR_). The results showed reduced information flow within the left hemisphere (lower D_LR_) and increased flow from the left to right hemisphere (higher I_LR_), as expected in patients with positive symptoms of schizophrenia. Statistically significant group differences were also found for the I_LR_ index, when CSD or AVERAGE was used, but the direction of information flow was in the opposite direction to what we expected.

## Discussion

### The influence of reference electrode on volume conduction effects

Comparison of the results obtained based on the original EEG data with those following CSD transformation and REST re-referencing revealed a reduced impact from common sources. This can be clearly seen in the adjacency matrices of every connectivity measure, particularly for bivariate measures such as PLV and PLI (c.f. Figs 1 and 4 for PLV, and Figs 2 and 5 for PLI). This is demonstrated by the reduction of PLV and PLI connections in the alpha band in the frontal part of brain (c.f. Figs 1 and 4 for PLV, and Figs 2 and 5 for PLI), as well as in the PLV connections between neighbouring electrodes (c.f. Figs 1 and 4). Moreover, in the adjacency matrices of DTF, the decrease in strength can be observed in an area near the reference electrode FCz (c.f. Fig 3). Concerns about the influence of volume conducted currents on the estimation of EEG-based connectivity measures have been raised by several investigators [54, 62–65]. Here, we show that the choice of reference electrode, in particular the application of CSD or REST, can reduce potential ‘contamination’ from common sources, i.e., volume conduction.

### Interactions between different frequency bands

Schizophrenia is a disorder characterized by functional brain reorganization in a frequency band-specific manner. Here, we examined each connectivity measure in each frequency band, separately. The largest group differences in connectivity were observed for the alpha band. The reduced alpha band synchronization in schizophrenic patients, compared with healthy controls, extended across the whole brain, as demonstrated by phase synchronization measures (PLV and PLI) when CSD or REST were used. A lower and more widespread synchronization has been reported previously [63]. In that study, the authors used non-partial and partial cross-correlations to examine brain connectivity in schizophrenia. Our results demonstrate that decreased synchronization is accompanied by increased information flow, quantified by DTF strength connectivity following CSD transformation.

Group differences were also found for other frequency bands. We found that the index strength evaluated using both phase synchronization measures decreased in delta band when REST was used. On the other hand, a stronger synchronization was observed in gamma band (PLV for REST and PLI for CSD). The strength of DTF decreased for lower frequency bands: delta (for CSD and AVERAGE), theta (for AVERAGE), and increased for higher frequency bands: alpha (for CSD), beta (for CSD) and gamma (for CSD and REST). DTF permits better differentiation using the data with CSD than other reference electrodes (AVERAGE or REST). This may be due to the fact that the information flows only from several sources (see rows in the adjacency matrices in Figs 3 and 6 and graphs for DTF in Figs 7 and 8), while the whole brain is involved in the synchronization process. Thus, CSD could be more informative in the case of localized changes in the brain, while AVERAGE or REST more appropriate for extensive activity.

Modulation of the amplitude of high-frequency oscillations by the phase of low-frequency oscillations is known as cross-frequency coupling [7–9]. It is hypothesized that low-frequency oscillations coordinate long-distance communication between distinct brain areas by modulating gamma oscillations within local assemblies. Thus, greater gamma synchronization, which can be observed in patients with schizophrenia, reflects an inability to properly modulate high-frequency oscillations by low-frequency oscillations, what may lead to cognitive impairment.

### The relationship between synchronization and information flow

In this paper, for the first time, directional connectivity measure (DTF) was assessed in connectivity analyses using the EEG data of patients with schizophrenia. DTF, in contrast to PLV and PLI, enabled the identification of the main sources and directions of information flow. In patients with schizophrenia, there was a greater number of flows from the posterior to the frontal brain areas. These results are in line with the results reported by other investigators [66], such as increased spectral power of spontaneous alpha activity in the posterior part of brain in schizophrenic patients.

We have shown that the greater number of flows corresponded to weaker PLI independently on the reference electrode (see graphs in Figs 7 and 8). Moreover, a comparison between DTF and PLV at alpha frequencies revealed that the target of information flow, in particular the frontal region, was an area of greater PLV when FCz, CSD or AVERAGE referencing procedures were used. Such relationship between PLV and DTF have been observed previously using EEG data obtained during eyes open and eyes closed resting state conditions [67]. Here, we found that the localization of area and strength of synchronization depends on the connectivity measure. PLV was greater in the frontal part of brain in patients with schizophrenia compared to healthy controls. On the other hand, PLI was smaller in the posterior part of brain. An exception was re-referencing using REST, for which the area of greatest synchronization, quantified by PLV, was located in the posterior part of brain. Another difference between REST and other re-referencing procedures was observed in source localization. The main sources were located in the occipital lobe for each electrode reference, excluding the REST, where they are located mainly in the parietal and central areas (see adjacency matrices in Fig 6 and graphs for DTF in Fig 8). A similar consequence of EEG data re-referencing using REST, i.e., the sources of alpha activity being located at parietal rather than occipital derivations, was observed in our previous study where we investigate the generation of alpha rhythm [42].

### Dependence of integration and segregation indices on threshold

Since other indices such as characteristic path lengths, clustering coefficients, and asymmetry measures depend on degree, their dependence on threshold was tested. A lower clustering coefficient and a shorter characteristic path length in the alpha band were found in the patients with schizophrenia compared to healthy persons for both measures of phase synchronization (PLV and PLI) applying CSD, AVERAGE or REST. This is consistent with findings reported by other groups examining brain connectivity in schizophrenia using EEG or MEG [35–36, 68–71]. A variety of linear and nonlinear estimators of connectivity, as well as graph-theory parameters, were used in these studies. However, these were all unidirectional measures, which do not allow for determination of causal relations between brain areas involved in the exchange of information.

### Fronto-posterior and inter-hemispheric asymmetry

Comparison of the asymmetry measures revealed group differences in fronto-posterior as well as inter-hemispheric asymmetry.

The results of PLV and DTF demonstrated group differences in fronto-posterior asymmetry following CSD transformation, while for PLI the differences were significant only when REST was used. Our results showed that increased synchronization, in patients with schizophrenia compared to healthy controls, was associated with fewer flows within the frontal areas and more flows from frontal to posterior brain areas. This finding support the idea of brain hypofrontality in schizophrenia [35–36, 70].

The only connectivity measure that revealed group differences, associated with inter-hemispheric asymmetry, was DTF calculated for REST. Our results showed reduced alpha band flow within the left hemisphere compared to the right hemisphere, and increased flow from the left to the right hemisphere in patients with schizophrenia. These results confirm the existence of an abnormal brain asymmetry, characterized by a reduction of leftward cerebral dominance [13–19] and impaired transfer of information from the right to the left hemisphere in schizophrenia [20], as well as an association between increased activation in the right hemisphere and positive symptoms characteristic of paranoid schizophrenia such as auditory hallucinations [25–26].

## Conclusions

By using data recorded in group of persons with schizophrenia and in a group of healthy controls, the present study highlights several methodological aspects related to the analyses of brain connectivity.

First, the observed connectivity patterns were dependent upon the choice of connectivity measure. Our results demonstrate the importance of examining functional brain connectivity by various measures for the same data. Each measure gives different information about the interactions, which occur within and between brain structures. Here, we analyzed three measures (PLV, PLI and DTF). These measures provide information about important features such as the phase synchronization (PLV, PLI) and directional flow of information between different structures of brain (DTF), which are subject to particular change in this disorder. The analysis of indices based on graph theory can be helpful for examining the neural mechanisms underlying disconnectivity disorders, by indicating the functional role of specific brain structures and brain rhythms in these processes. However, the exact localization of sources as well as the synchronization pattern depend on the choice of reference electrode.

Second, choice of reference electrode has an impact on the estimation of connectivity measures, and consequently, on the estimation of indices based on graph theory and the differentiation between groups or, more generally, between different brain states. There is not an ideal reference electrode; the choice of reference electrode depends on the specific situation. An ideal reference electrode should be placed as far away as possible from the source of brain activity. For this reason, the REST re-referencing procedure has been introduced. As we expected, the correlation between CSD and REST was greater than the correlation between CSD and another reference electrode. This was particularly clear in the strength of connections in the alpha band for each connectivity measure. Moreover, the CSD transform as well as the REST reduced effects of volume conduction. In some cases the REST was better than CSD, particularly in the evaluation of inter-hemispheric asymmetry by DTF or fronto-posterior asymmetry by PLI.

Finally, different brain states as well as brain disorders, particularly schizophrenia, are frequency-dependent. Thus, the connectivity measures should be analyzed in each frequency band, separately.

The purpose of the present study was to provide an overview of the various methodological aspects that may influence the results of connectivity studies. Nevertheless, this study has some limitations. The small number of EEG channels precluded us from performing source level analysis. Moreover, future studies in larger patient groups are warranted to evaluate the correlation between the indices based on graph theory and characteristics of schizophrenia, such as behavior scale scores and illness duration.
